# Mycophagous Mite, *Tyrophagus putrescentiae,* Prefers to Feed on Entomopathogenic Fungi, except *Metarhizium* Generalists

**DOI:** 10.3390/microorganisms12061042

**Published:** 2024-05-22

**Authors:** Cuiyi Ou, Qichun Chen, Xiangyu Hu, Yuhao Zeng, Ke Zhang, Qiongbo Hu, Qunfang Weng

**Affiliations:** National Key Laboratory of Green Pesticide, College of Plant Protection, South China Agricultural University, Guangzhou 510642, China; ocy@stu.scau.edu.cn (C.O.); cqc@stu.scau.edu.cn (Q.C.); xiangyu0500@163.com (X.H.); 13533061123@163.com (Y.Z.); zhangk@scau.edu.cn (K.Z.)

**Keywords:** *Tyrophagus putrescentiae*, feeding preference, entomopathogenic fungi, *Metarhizium*

## Abstract

(1) Background: The mycophagous mite, *Tyrophagus putrescentiae*, was found to feed on entomopathogenic fungi (EPF) in our previous experiments, which seriously impacted the culture and preservation of fungal strains. Therefore, it is necessary to investigate the biological characteristics of the occurrence and damage to EPF. (2) Methods: The mite’s growth and development and feeding preference were surveyed by comparative culture and observation; also, optical and electronic microscopies were employed. (3) Results: *T. putrescentiae* could survive normally after being fed on seven EPF species, including *Purpureocillium lilacinum*, *Marquandii marquandii*, *Cordyceps fumosorosea*, *Beauveria bassiana*, *Metarhizium flavoviride*, *Lecanicillium dimorphum*, and *Metacordyceps chlamydosporia*. The first four fungi were the mite’s favorites with their greater feeding amount and shorter developmental duration. Interestingly, the mite could also feed on *Metarhizium anisopliae* and *Metarhizium robertsii*, but this led to the mite’s death. After feeding on *M. anisopliae* and *M. robertsii*, the mites began to die after 24 h, and the mortality rate reached 100% by 72 h. Observation under optical microscopy and scanning electron microscopy revealed that the conidia of *M. anisopliae* and *M. robertsii* adhered to the mite’s surface, but there was no evidence of penetration or invasion. However, dissection observation indicated that the two *Metarhizium* species germinate and grow within the mite’s digestive tract, which implies that *Metarhizium* generalists with broad-spectrum hosts and the production of destruxins have acaricidal activity toward the mycophagous mites.

## 1. Introduction

*Tyrophagus putrescentiae* (Acarina: Acaridae) is one of the most important stored food pests, with a worldwide distribution [1]. It is small in size and reproduces rapidly. Especially under suitable temperature and humidity conditions, its population grows swiftly, which makes it prone to outbreaks and causes significant harm [2]. The mite not only damages food storage but also impacts human health. It contains allergens capable of inducing IgE-mediated allergic reactions in individuals with a genetic predisposition [3,4]. It is recognized as an important allergenic mite that can cause allergic diseases and harm to humans through inhalation or skin contact, including conditions such as allergic asthma, allergic dermatitis, pulmonary acariasis, and intestinal acariasis [5,6].

This mite’s predilection for fungi has been widely reported. It has the ability to feed on an array of fungal species, encompassing those of medical and agricultural significance, as well as various molds and yeasts, including *Fusarium*, *Aspergillus*, *Candida*, *Hyphopichia*, *Penicillium*, *Rhizophus*, *Trichophyton*, etc. [7,8]. The mite feeds on fungal spores and hyphae for their development and reproduction and assists in the dispersal of fungi, with fungal spores being spread through their bodies and undigested spores excreted in feces [7,9]. Fungal hyphae, which consist of cell walls abundant in chitin and cells containing trehalose, are a source of rich lipids and proteinaceous substances, which provide a nutritious diet for the mite [10,11,12]. When mites ingest these hyphae, their digestive tracts secrete trehalase, an enzyme capable of breaking down the cellular contents of the hyphae to digest fungi [13,14]. Consuming different types of fungi can potentially influence the mites’ feeding preferences, growth rate, reproductive capacity, and lifespan [15].

Entomopathogenic fungi (EPF) are the main pathogens that balance populations of insects in nature. More than 1000 species of EPF have been discovered, which are mainly distributed in the order Hypocreales in the phyla Ascomycota, which includes many famous EPF genera such as *Beauveria*, *Metarhizium*, *Cordyceps*, *Lecanicillium*, *Purpureocilium*, etc. (https://www.mycobank.org/, accessed on 20 April 2024), as well as a small number of species distributed in Entomophthoromycota, Blastocladiomycota, Microsporidia, and Basidiomycota [16,17]. The EPFs *B. bassiana*, *M. anisopliae*, *M. robertsii*, *C. fumosorosea*, *C. javanica*, *L. muscarium,* and *P. lilacinum* have been registered as pesticides worldwide and are used to control insect, mite, and nematode pests [18]. For example, *B. bassiana* and *M. anisopliae* are registered as pesticides in 48 products in China to control moths, beetles, aphids, locusts, flies, etc. (http://www.chinapesticide.org.cn/, accessed on 20 April 2024). Also, they can control the spider mites, including the European red mite, *Panony chusulmi* (Acari: Tetranychidae), *Tetrany chusurticae* (Koch) (Acari: Tetranychidae), etc. [19,20]. 

*Metarhizium*, as a type of entomopathogenic fungus, is widely distributed in nature and can control more than 200 species of pests, mites, and nematodes [21]. *Metarhizium* typically infects insects through their cuticle, but it may also infect the digestive tract or respiratory systems [22]. *Metarhizium* generalists can infect a wide spectrum of host insects, whereas the specialists have a narrow host range. Generalist species, including *M. anisopliae*, *M. robertsii*, and *M. brunneum*, can infect a variety of insects. Moreover, *M. acridum* is a specialist with a narrow insect host range restricted to Orthoptera (grasshoppers, locusts, or crickets). *Metarhizium* speciation shows divergence from specialist species (*M. acridum*) to transitional species with intermediate host ranges (*M. guizhouense*), followed by generalist species (*M. robertsii*) [23].

In our previous experiments, we found that the mite feeds EPFs and influences fungal culture and preservation. Because there have not been reports about the interaction of EPFs and *T. putrescentiae*, we carried out the current research. The study is intended to compare the responses of *T. putrescentiae* feeding on various entomopathogenic fungi, including *M. anisopliae*, *M. robertsii*, *M. flavoviride*, *C. fumosorosea*, *B. bassiana*, *P. lilacinum*, *L. dimorphum*, *Marquandii marquandii,* and *Metacordyceps chlamydosporia*.

## 2. Materials and Methods

### 2.1. Population of Tyrophagus putrescentiae and Maintenance

The population of *T. putrescentiae* was maintained by our group. The mites were fed with yeast powder and cultivated in an incubator at (26 ± 1) °C with an RH of (85 ± 10)%. The mites with similar sizes were selected to be used in subsequent experiments. The mites were separately transferred to media with the nine EPF strains provided in this experiment and bred for more than five generations to construct the experimental populations. The mites used in this experiment were non-sexual.

### 2.2. Entomopathogenic Fungi Strains and Culture

The nine EPF strains tested in this study were isolated from the soil of Guangxi Province, China, and stored by our group [24]. Each strain’s slant was inoculated onto the PDA (Potato Dextrose Agar) plate and cultured at (26 ± 1) °C and an RH of (85 ± 10)% for about 7 days for further use. The EPF strains were listed in Table 1.

### 2.3. Feeding Preference of T. putrescentiae on Entomopathogenic Fungi

There were three tests to evaluate the mite’s feeding preference, i.e., feeding amount in non-choice, chemotaxis index in choice, and attracting number. The software SPSS 26.0 (IBM, Armonk, NY, USA) was used to analyze the data.

For the feeding amount in the non-choice test, a disk (diameter 5 mm) of EPF hyphae was picked out from a PDA plate and transferred to the center of the new PDA plates. Once the fungus had grown to cover half of the plate, fifty adult mites of consistent size were selected and transferred onto the plate. Seal the Petri dishes with parafilm to prevent the mites from escaping. The culture conditions were the same as in Section 2.2. The experiments were replicated three times. After the mites were introduced for 7 and 14 days, the plates were photographed. The feeding amounts (area) were evaluated based on the picture analysis by employing the software ImageJ (V1.8.0, National Institutes of Health, Stapleton, NY, USA) [25]. 

For the chemotaxis index in choice test, a four-point chemotaxis experiment [25] was conducted to evaluate the host preferences of the mite to different EPFs. A PDA plate (diameter = 9 cm) was divided into four quadrants (I, II, III, and IV), and nine EPF species were grouped, with three species per group (Table 2) and IV as the blank control. The 5 mm disks of different EPFs were placed in the three quadrants of the PDA plate in a predetermined order, while a sterile PDA disk was placed in the fourth quadrant as a blank control. Twenty adult mites of consistent size were placed in the center of the plate. Then, they were subjected to the same treatment as above. The experiment was repeated three times. After 12 h, the number of mites in each quadrant was counted, and the chemotaxis index (CI) for each group was calculated according to Equation (1).

Quadrant IV is the blank control.

CI = (Ntr − Nck)/Ntol(1)

where Ntr is the number of mites in quadrants; Nck is the number of mites in the blank control quadrant; and Ntol is the total number of mites in the four quadrants.

For the attracting number test, the hypha disks of the nine EPFs were placed at equal distances on a PDA plate, with each dish containing 500 mites of consistent size. The dish was sealed with parafilm and placed in an incubator at the same temperature and light conditions as described above. After 24 h of free feeding, the number of mites on each fungal disk was counted, and the attraction rate was calculated. Each treatment was repeated three times.

AR (%) = 100 × NF/ND(2)

where AR is the attraction rate; NF is the number of mites attracted to each fungal strain; and ND is the total number of mites attracted to each disk.

### 2.4. Developmental Duration of T. putrescentiae Fed on Different Entomopathogenic Fungi

The hypha disks with a diameter of 5 mm were transferred onto the PDA plate (60 × 15 mm). Then, the female adult mites that have been feeding on different EPFs were transferred to a centrifuge tube for oviposition for 12 h, and the eggs were collected. The eggs were transferred to the corresponding EPF’s plate (the same EPF strain as their parent’s feeding). One egg was introduced on each plate, and a total of 30 eggs were observed for each strain. For data collection, we observed them once every 12 h and recorded the hatching status of the eggs as well as the developmental duration of each mite stage until they reach the adult stage. At each stage, photos were taken.

### 2.5. Bioactivity of M. anisopliae and M. robertsii on T. putrescentiae

#### 2.5.1. Bioassay of *Metarhizium* spp. against Mites

Twenty adult mites of uniform size were placed individually into an EPF PDA plate. We allowed them to feed on the fungi freely, then sealed the containers with parafilm and cultured them as above. We observed and recorded the status of the mites under a stereomicroscope every 12 h. We considered mites that do not move when touched with a brush dead, recorded the number of dead mites, and took photographs of the infected mites. *B. bassiana* was used as a control treatment, with each treatment being repeated four times.

#### 2.5.2. Infection Observation of *Metarhizium* spp. on Mites

During microscopy observations, we selected individual mites and placed them separately into plates inoculated with *M. anisopliae* and *M. robertsii* strains. We monitored the mites’ statuses at 12-hour intervals (12 h, 24 h, 36 h, and 48 h) following their exposure to two species of *Metarhizium*. A stereomicroscope (Yuehe, YZ38, Shanghai Yuehe Biotech Co., Ltd., Shanghai, China) was used to identify and pick out mites that appear inactive or are dead (and show no surface mycelium growth) for dissection. Subsequently, the internal tissue conditions of these mites were examined under a microscope (Phenix, BMC512-IPL, Phoenix Optical Group Co., Ltd., Shanghai, China), and the photographs were captured for documentation purposes.

During the scanning electron microscopy procedure, from the plates inoculated with *M. anisopliae* and *M. robertsii* strains on which the mites have been feeding, we selected mites in the early stages of infection (not yet dead, exhibiting inactive behavior, capable of movement when gently prodded with a brush, and showing no surface mycelium growth) for sample preparation. We placed the collected mites in a 2.5% glutaraldehyde solution for fixation for 24 h, then washed them three times with 0.1 mol/L PBS buffer, 10 min per wash. Subsequently, dehydration of the mites was performed using a series of ethanol concentrations, i.e., 30%, 50%, 70%, 80%, and 90%, each for 10 min, followed by two rounds of dehydration with 100% ethanol, also for 10 min each. After dehydration, we proceeded with drying, then removed the samples and applied a gold sputter coating. Once this process was completed, photographs were captured using a scanning electron microscope.

## 3. Results

### 3.1. Feeding Preference of T. putrescentiae

**Figure 1:**
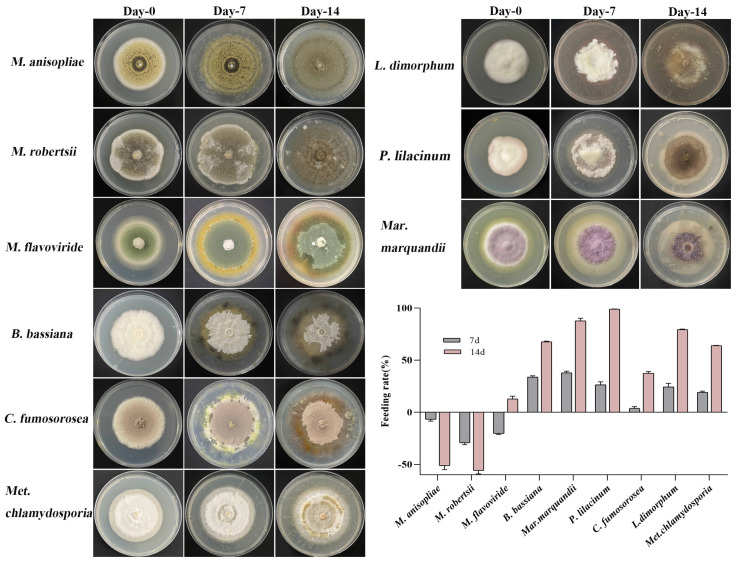
Feeding amount of *T. putrescentiae* on entomopathogenic fungi. Fifty adult mites were introduced into the PDA plate when the fungus colonized half of the dish. The experiments were replicated three times. After the mites were introduced for 7 and 14 days, the plates were photographed. The feeding rate is based on the amount (area) by employing the software ImageJ(×64)1.8.0. Culture conditions were set at (26 ± 1) °C and an RH of (85 ± 10)%.

It was found that the mite feeds EPFs in different amounts (Figure 1). Generally, the mite rapidly consumed *P. lilacinum*, *Mar. marquandii*, and *L. dimorphum* with feeding rates of 99.04%, 87.65%, and 79.44% within 14 days post-treatment. However, when feeding on *M. flavoviride*, *B. bassiana*, and *C. fumosorosea*, the mites had a lower feeding rate of 15−65% in 14 days. Notably, *M. flavoviride* exhibited a negative feeding rate in the first 7 days, showing rapid fungal growth that outpaced the consumption by the mites. After the 7th day, with the fungal hyphae continuously growing and producing spores, the mite population began to increase, and the feeding amounts significantly increased by the 14th day, with a feeding rate of 12.85%. However, the mites consumed *M. flavoviride,* apparently slower than other strains. When feeding on *Met. chlamydosporia*, the fungal strains continued to grow after 14 days, with a feeding rate of 64.06%. The observed mite population was small, as most of the mites had escaped. For *M. anisopliae* and *M. robertsii*, the damage caused by the mites was minimal, and the population of mites feeding on these strains continuously decreased.

**Figure 2:**
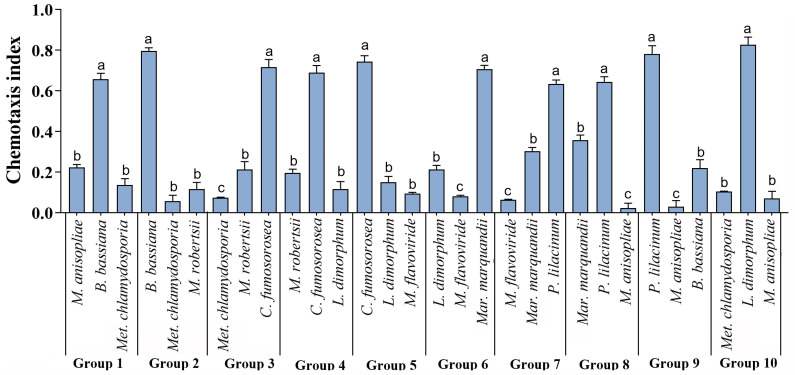
Chemotaxis Index of *T. putrescentiae* to different EPFs. A four-point chemotaxis experiment was conducted in a choice test. A PDA plate (diameter = 9 cm) was divided into four quadrants, and nine EPF species were grouped, with three species per group. The 5 mm disks of different EPFs were placed in the three quadrants of the PDA plate in a predetermined order, while a sterile PDA disk was placed in the fourth quadrant as a blank control. Twenty adult mites of consistent size were placed in the center of the plate. The experiment was repeated three times. After being cultivated for 12 h, the number of mites in each quadrant was counted, and the chemotaxis index (CI) for each group was calculated and checked by LSD. The different letters above each column indicate a significant difference (*p* < 0.05).

Through the four-point chemotaxis preference test, the differences in the chemotactic values towards each fungal species by *T. putrescentiae* were significant (Figure 2). Among the different groups from Group 1 to Group 10, the highest CI (Chemotaxis Index) values were observed for *B. bassiana*, *C. fumosorosea*, *Mar. marquandii*, *P. lilacinum*, and *L. dimorphum*, all with CI values greater than 0.60. In Group 7, the CI value for *P. lilacinum* was higher than that for *Mar. marquandii*, with values of 0.63 and 0.30, respectively. In Group 10, the CI value for *L. dimorphum* was higher, and when *L. dimorphum* was combined with *C. fumosorosea* and *Mar. marquandii*, the CI values were below 0.3. Therefore, from the four-point chemotaxis preference test, it can be concluded that mites have a stronger preference for feeding on *P. lilacinum*, *Mar. marquandii*, *B. bassiana*, and *C. fumosorosea*.

**Figure 3:**
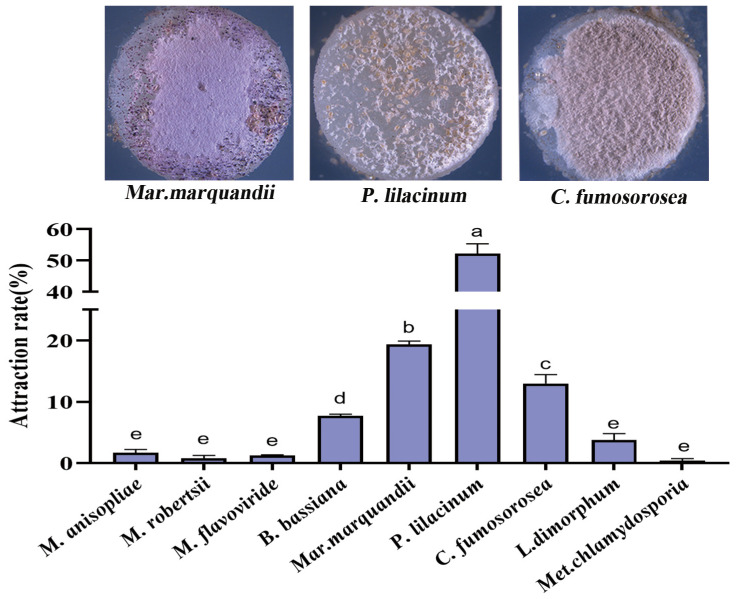
Attraction of *T. putrescentiae* by different EPFs. The hypha disks of the nine EPFs were placed at equal distances in a PDA plate, and the 500 mites with consistent size were introduced in each dish. Each treatment was repeated three times. After 24 h of free feeding, the number of mites on each fungal disk was counted, and the attraction rats were evaluated and checked by LSD. The different letters above each column indicate a significant difference (*p* < 0.05).

The attraction effect of EPF on the mites was observed (Figure 3). The results revealed that *P. lilacinum* had the highest attraction rate of 52.17%, followed by *Mar. marquandii* with 19.32%, *C. fumosorosea* with 12.97%, and *B. bassiana* with 7.68%. Additionally, the attraction rates of *M. anisopliae*, *M. robertsii*, *L. dimorphum*, and *Met. chlamydosporia* were all below 4%, with *Met. chlamydosporia* having the lowest attraction rate of only 0.36%. Overall, the results suggested that *T. putrescentiae* has a strong preference for feeding on *P. lilacinum* and *Mar. marquandii*.

### 3.2. Development Duration of T. putrescentiae Fed on Different Entomopathogenic Fungi

**Figure 4:**
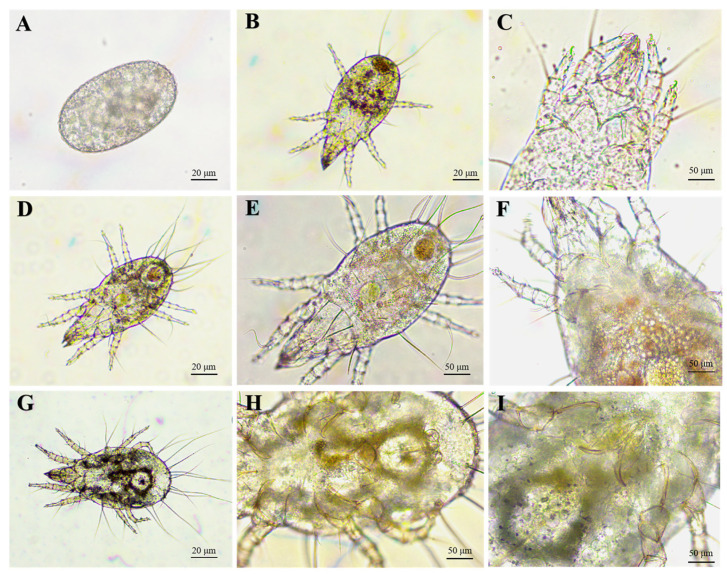
Morphology of *T. putrescentiae* in different developmental stages. Observation under a stereoscopic microscope. The mite had several stages in a life cycle of about ten days, i.e., egg → larva → quiescence → first instar nymph → quiescence → second instar nymph → quiescence → adult. (**A**): egg; (**B**): larva; (**C**): larva forepart, arrow indicates the coxa; (**D**): nymph; (**E**): back of nymph; (**F**): nymph forepart; (**G**): adult; (**H**): anal area of male adult; (**I**): genital area of female adult.

When fed on the seven EPFs, *T. putrescentiae* had the same stages in a life cycle of about ten days, i.e., egg → larva → quiescent larva → first instar nymph → quiescent nymph → second instar nymph → quiescence → adult (Figure 4). The larvae were similar to nymphs and adults in shape, but the larvae had three pairs of legs, whereas nymphs and adults had four pairs of legs. The genitalia were underdeveloped in larvae, developing but not yet distinct in nymphs, and distinct fan-shaped in adults (Figure 4).

However, the mite exhibited different durations from egg to adult when fed on various EPFs (Table 3). The total duration ranged from 9–12 d. The shortest life cycle was recorded as 9.57 d in feeding on *Mar. marquandii*, followed by 10.08 d in *P. lilacinum* treatment. The longest duration, i.e., 11.97 d, was observed in feeding on *Met. chlamydosporia*. The shortest egg stage was 3.14 d in *Mar. marquandii*, and the longest egg stage is 3.71 d in *M. flavoviride*. The longest and shortest larvae stages of 1.99 d and 1.34 d were respectively recorded in *Met. chlamydosporia* and *C. fumosorosea*.

### 3.3. The Biological Activity of Metarhizium against T. putrescentiae

#### 3.3.1. Acaricidal Activity

**Figure 5:**
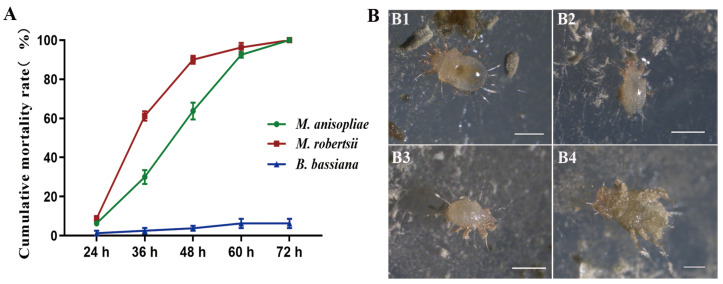
Acaecidal activity of EPFs against *T. putrescentiae*. Twenty adult mites of uniform size were placed individually into each PDA plate with EPF. The culture conditions were (26 ± 1) °C and an RH of (85 ± 10)%. The status of the mites under a stereomicroscope was observed and recorded every 12 h. The mites that do not move when touched with a brush are considered dead; the number of dead mites was recorded; and photographs of the infected mites were taken. *B. bassiana* was used as a control treatment, with each treatment being repeated four times. (**A**) Mortality of mites fed with *M. anisopliae*, *M. robertsii,* and *B. bassiana*. (**B**) Symptoms of *T. putrescentiae* infected with *Metarhizium*. (**B1**): Normal feeding mites. (**B2**): Mites that are inactive after feeding for 24 h. (**B3**): Mite dead for 36 h. (**B4**): The abdomen of the dead mite for 36 h. Scale, 1 mm.

Obviously, *M. anisopliae* and *M. robertsii* had acaricidal activity against the mites (Figure 5). Mites began to die at 24 h after consuming the two fungi, with a mortality rate reaching 100% by 72 h. It seemed that *M. robertsii* has higher toxicity to mites, with cumulative mortality of 61.25%, 90.0%, and 100% at 36 h, 48 h, and 72 h after treatment, respectively, which were all higher than that of *M. anisopliae* (Figure 5A). However, the mites infected by *M. anisopliae* and *M. robertsii* showed the same symptoms (Figure 5B). On the contrary, *B. bassiana* had few lethal effects on the mites.

#### 3.3.2. Infection Progress of *Metarhizium* on *T. putrescentiae*

**Figure 6:**
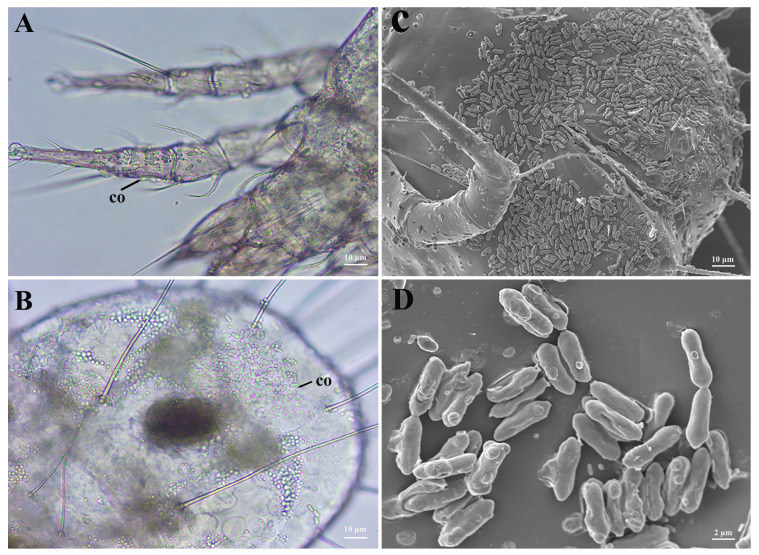
Observation of *T. putrescentiae* infected by *M. anisopliae*. (**A**,**B**): Microscope profiles of mites treated after 12 h; co indicate conidia. (**C**,**D**): SEM profiles of the mites treated after 24 h indicate conidia adhere to the mite cuticle but do not germinate.

The same infection progress was observed in the two species of *Metarhizium* on *T. putrescentiae* under microscopes. At the early infection, the fungal conidia adhered to the mite’s surfaces were clearly found at 12 h after treatment (Figure 6A,B), while a large number of conidia within the digestive tract were observed, indicating that the mites fed conidia of *Metarhizium*. Furthermore, under the SEM, it was found that lots of conidia adhered on the mite’s surface at 24 h after treatment, but no structures of germination or invasion were observed, which suggested that the two species of *Metarhizium* might not infect through penetrating the mite’s cuticle (Figure 6C,D).

**Figure 7:**
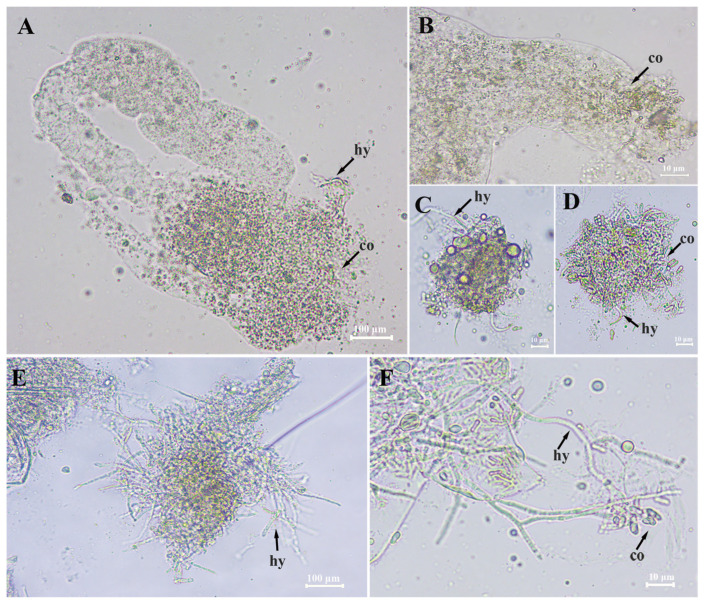
Microscope profiles of the digestive tract of *T. putrescentiae* treated by *M. robertsii* after 24 h (**A**–**D**) and 36 h (**E**,**F**). (**A**): digestive tract; (**B**): midgut; (**C**,**D**): germinating spores and hyphae in the digestive tract. (**E**,**F**): germinating spores and hyphae in the digestive tract of mites treated after 36 h. co: conidia, hy: hypha.

To determine the two species of *Metarhizium* that infect *T. putrescentiae* through the digestive tract, the mites in the early stage of disease (after 24 h of treatment) and starting to die (36 h after treatment) were dissected and observed under a microscope (Figure 7). It was found that *Metarhizium* conidia can germinate and infect mites. At 24 h after treatment, a large number of conidia were observed in the digestive tract of the disease mites (they had not died yet) (Figure 7A–D), and in the punctured tract, the germinated spores and hyphae were clearly found. After 36 h of treatment, the dissection observation revealed that a large number of spores and hyphae were released from the mite digestive tract after it was punctured (Figure 7E,F). Within 12 h after the mite died, the spores inside the body continued to germinate and grow, eventually filling the entire body cavity with hyphae. The results strongly suggest that the digestive tract is an important way for infecting *M. anisoplieae* and *M. robertsii* on the mite.

## 4. Discussion

This research first reports that the mycophagous mite, *T. putrescentiae,* feeds on EPFs, preferring *P. linacinum*, *Mar. marquandii*, *C. fumosorosea*, and *B. bassiana* to *M. flavoviride*, *Mar. marquandii*, *L. dimorphum*, and *Met. chlamydosporia*, while ding from feeding the *Metarhizium generalists*, *M. anisopliae,* and *M. robertsii*. It shows the significance of EPF’s research and application, as well as the mite’s control. For example, it is necessary to prevent mite contamination in the processes of EPF production and experimentation. Also, it is an interesting topic that uses *M. anisopliae* and *M. robertsii* to control the mite, although many further experiments are required. 

The mite feeds a wide range of fungi, but there is preference; those that contain rich proteins and carbohydrates (for example, *Flammulina velutipes*) are in its favor; also, the volatilized terpene compounds of the fungi are more attractive for the mite [26,27]. This study found that *T. putrescentiae* is attracted to EPFs with an apparent chemotaxis. Indeed, EPFs can attract some insects, such as aphids and ants, by emitting some volatiles [28,29]. *M. brunneum* produces the volatile organic compounds 1-octen-3-ol and 3-octanone, which proved to be attractive to the entomopathogenic nematode *Heterorhabditis bacteriophora* and impact the insects *Galleria mellonella*, *Cydia splendana,* and *Curculio elephas* [30]. What substances produced by the preferred EPFs, such as *P. lilacinum*, *Mar. marquandii*, *C. fumosorosea*, and *B. bassiana*, can attract mites? How do EPFs such as *M. anisopliae*, *M. robertsii*, *L. dimorphum*, *Met. chlamydosporia,* and *Met. chlamydosporia* repel mites? These questions need to be elucidated further.

The growth and development duration of *T. putrescentiae* is greatly influenced by the type of food, which may be related to the mite’s ability to digest fungal material [31]. It was discovered that the type of food affects the mite’s oviposition period, post-oviposition period, and lifespan [32]. This experiment also found that *T. putrescentiae* exhibits significant differences in growth duration when feeding on different EPFs. When feeding on *Mar. marquandii*, the mite has the shortest development duration with 9.57 d, while feeding on *Met. chlamydosporia* has the longest development period with 11.97 d. The trehalase in the digestive tract of mites can digest trehalose in the fungal hyphae, and the digestive capacity varies when feeding on different fungi. The better the mite’s ability to digest food, the faster the population grows [33]. Furthermore, whether a fungus can be consumed by mites is related to the presence of fungal toxins, secondary metabolite content, and the availability of alternative food sources. 

Interestingly, it was found that in this study, the mite dies from feeding *M. anisopliae* and *M. robertsii*. These two EPFs are considered the generalist *Metarhizium*, which has broad host spectra in relation to the specialist *M. acridum* with the single host. The generalist *Metarhizium* acquired destruxins, a cyclopeptidic mycotoxin, which is an insecticidal compound with multiple bioactivities such as anti-immunity, anti-feeding, and growth regulation [25,34,35]. The results suggest that the two *Metarhizium* species primarily cause insect disease through the digestive tract. However, there has been considerable debate about whether EPFs can infect insects through the digestive tract, because the most evidence supports the idea that EPFs infect insects by penetrating the cuticle. The digestive tract environments of different insects vary, and factors such as excessive acidity or alkalinity, as well as temperature and humidity, can inhibit spore germination. The successful germination of spores is crucial for further infection, although there are a few reports of *Metarhizium* infection in insects through the digestive tract [36]. In this study, germinating *Metarhizium* spores were found in the intestines of *T. putrescentiae* in the early stages of the disease. It is rational that *Metarhizium* secretes destruxins into the mite’s digestive tube, which leads to mite death. In this study, another *Metarhizium* species, *M. flavoviride*, showed lower preference for the mite, but it gives the mite a shorter duration of 11.06 d and does not lead to mite death. This fungus is considered the specialist *Metarhizium,* with far fewer amounts and types of DTXs than the generalists [37]. Obviously, *M. flavoviride* differs from *M. anisopliae* and *M. robertsii* in that it can provide the necessary nutrients for the growth and development of the mite.

## 5. Conclusions

The results show that the mite, *T. putrescentiae*, prefers the EPFs, *Mar. marquandii*, *C. fumosorosea*, *P. lilacinum*, and *B. bassiana* to *C. fumosorosea*, *L. dimorphum*, *M. flavovirid*, and *Met. chlamydosporia*. However, when the mite feeds on *M. anisopliae* and *M. robertsii*, they will die in 72 h. The microscopic observation indicates that the mites died from the fungal infection through conidia germination in the mite’s digestive tract. It suggests that the generalist *Metarhizium* secretes multiple insecticidal toxins, destruxins, so that fungi can invade the digestive tract. This study provides new insights into the feeding and bioactivity of *T. putrescentiae*, which can be used for its control.

## Figures and Tables

**Table 1:** Strain information tested in this experiment.

Strain No.	Species
MaGX3701	*Metarhizium anisopliae*
MrGX0603	*Metarhizium robertsii*
MfGX33Y01	*Metarhizium flavoviride*
BbGX6502	*Beauveria bassiana*
PiGX19S01	*Purpureocillium lilacinum*
MmGX4202	*Marquandii marquandii*
CfGX4206	*Cordyceps fumosorosea*
LdGX4702	*Lecanicillium dimorphum*
McGX08A01	*Metacordyceps chlamydosporia*

**Table 2:** The EPF group of the chemotaxis index in the choice test.

Quadrant	I	II	III
Group 1	*M. anisopliae*	*B. bassiana*	*Met. chlamydosporia*
Group 2	*B. bassiana*	*Met. chlamydosporia*	*M. robertsii*
Group 3	*Met. chlamydosporia*	*M. robertsii*	*C. fumosorosea*
Group 4	*M. robertsii*	*C. fumosorosea*	*L. dimorphum*
Group 5	*C. fumosorosea*	*L. dimorphum*	*M. flavoviride*
Group 6	*L. dimorphum*	*M. flavoviride*	*Mar. marquandii*
Group 7	*M. flavoviride*	*Mar. marquandii*	*P. lilacinum*
Group 8	*Mar. marquandii*	*P. lilacinum*	*M. anisopliae*
Group 9	*P. lilacinum*	*M. anisopliae*	*B. bassiana*
Group 10	*Met. chlamydosporia*	*L. dimorphum*	*M. anisopliae*

**Table 3:** The growth duration of *T. putrescentiae* feeding on different entomopathogenic fungi.

EPF	Mite Developmental Duration (d)							
	Egg	Larvae	Quiescence	First Instar Nymph	Quiescence	Second Instar Nymph	Quiescence	Total
*M. flavoviride*	3.71 ± 0.05 a	1.94 ± 0.07 a	0.65 ± 0.04 cd	1.69 ± 0.03 b	0.58 ± 0.04 c	1.75 ± 0.06 ab	0.75 ± 0.05 c	11.06 ± 0.07 b
*B. bassiana*	3.65 ± 0.07 a	1.54 ± 0.06 c	0.54 ± 0.03 d	1.33 ± 0.05 d	0.63 ± 0.04 bc	1.58 ± 0.04 bc	0.86 ± 0.05 abc	10.13 ± 0.10 cd
*P. lilacinum*	3.50 ± 0.09 ab	1.80 ± 0.08 b	0.73 ± 0.06 bc	1.52 ± 0.08 bc	0.74 ± 0.05 ab	1.72 ± 0.05 ab	0.79 ± 0.05 bc	10.08 ± 0.17 cd
*Mar. marquandii*	3.14 ± 0.05 c	1.64 ± 0.04 bc	0.61 ± 0.03 cd	1.08 ± 0.04 e	0.58 ± 0.04 c	1.73 ± 0.04 ab	0.79 ± 0.05 bc	9.57 ± 0.08 d
*C. fumosorosea*	3.62 ± 0.05 a	1.34 ± 0.04 d	0.64 ± 0.04 cd	1.32 ± 0.06 d	0.80 ± 0.05 a	1.83 ± 0.07 a	0.86 ± 0.05 abc	10.42 ± 0.14 c
*L. dimorphum*	3.34 ± 0.07 b	1.65 ± 0.05 bc	0.83 ± 0.06 ab	1.35 ± 0.06 cd	0.70 ± 0.05 abc	1.52 ± 0.05 c	0.93 ± 0.03 ab	10.34 ± 0.09 c
*Met. chlamydosporia*	3.57 ± 0.08 a	1.99 ± 0.07 a	0.91 ± 0.03 a	1.95 ± 0.09 a	0.81 ± 0.04 a	1.73 ± 0.04 ab	1.00 ± 0.07 a	11.97 ± 0.13 a

The hypha disks with a diameter of 5 mm were transferred onto the PDA plate (60 × 15 mm). One egg of mite was introduced into each PDA plate, and a total of 30 eggs were repeated in each EPF. The culture conditions were (26 ± 1) °C and an RH of (85 ± 10)%. For data collection, the hatching status of the eggs as well as the developmental duration of each mite stage were recorded until they reached the adult stage. The values were presented as the mean ± SE. Means within a column followed by the same letter are not significantly different (LSD test: *p* < 0.05).

## Data Availability

Data are contained within the article.
